# Fooled by Temperature

**Published:** 1881-09

**Authors:** 


### "Fooled by Temperature."

In his little book on Semiology, lately published, Dr. J. Milner Fothergill has this passage: Often a rise or fall in the temperature heralds a coming change, of which it may be the first outward sign. On the other hand, the student must know that at times rapid rises of temperature are nervous in origin, are, in fact, "true neuroses." In one case which came under my notice, in a very nervous girl, for months the temperature, when taken, was over 103°. This rise was accompanied by increased rapidity in the respiration and the pulse. Yet she was sinking of inanition, and never approached the typhoid condition which is the consequence of a sustained high temperature, nor gave any indication of persisting fever. Once the temperature, when taken, was 104°, yet she was not at all "feverish;" it was just excitement, and too evanescent to produce any distinct consequences. Further, listen to what Austin Flint says: "The physician is liable to be misled by placing too much reliance on the laws of temperature. They are not infrequently interfered with by complications and accidental events. As an illustration, a young girl had passed through typhoid fever, convalescence being declared, in connection with other symptoms, by the laws of thermometry belonging to the decline of fever or defervescence in this disease. Suddenly hysterical symptoms were manifested, and the temperature rose to 105°. The physician, a man of learning and large experience, was naturally alarmed. In a few hours, however, the temperature declined, and recovery took place without further impediment. The expressive comment made by the physician was: "This is not the first time I have been fooled by temperature!" With regard to the information furnished by the thermometer, as well as other diagnostic symptoms, it is to be borne in mind that there are exceptions to rules which are generally applicable. It is in the female sex that these neurosal disturbances are usually manifested. At the catamenial week of the menstrual cycle, temperature perturbations are common, and a pyrexia, for which there is no apparent cause, may at these times cause unnecessary alarm. *Experte credo!—Pacific Medical and Surgical Journal.*

